# Magnetic resonance imaging of the cranial nerves in congenital, traumatic, and vascular diseases: a pictorial essay

**DOI:** 10.1590/0100-3984.2020.0039

**Published:** 2021

**Authors:** Mariana Dalaqua, Felipe Barjud Pereira do Nascimento, Larissa Kaori Miura, Fabiano Reis, Márcio Ricardo Taveira Garcia, Alcino Alves Barbosa Júnior

**Affiliations:** 1 Department of Diagnostic and Interventional Imaging, Hôpital du Valais, Sion, Valais, Switzerland.; 2 Imaging Department, Hospital Israelita Albert Einstein, São Paulo, SP, Brazil.; 3 Department of Radiology, Universidade Estadual de Campinas (Unicamp), Campinas, SP, Brazil.; 4 Diagnósticos da América S/A (DASA), Barueri, SP, Brazil.

**Keywords:** Neuroimaging/methods, Radiology/methods, Magnetic resonance imaging/methods, Cranial nerves/diagnostic imaging, Neuroimagem/métodos, Radiologia/métodos, Ressonância magnética/métodos, Nervos cranianos/diagnóstico por imagem

## Abstract

The cranial nerves, which represent extensions of the functional structures of the brain, traverse the head and neck. They are connected to various cranial structures and are associated with several diseases. An in-depth understanding of their complex anatomy and normal imaging appearance allows the examiner to identify and characterize abnormalities with greater precision. One important tool for evaluating the cranial nerves is contrast-enhanced magnetic resonance imaging, especially three-dimensional steady-state free precession sequences, which provide high soft-tissue and spatial resolution, despite the slenderness of the nerves. In most cases, imaging findings are nonspecific. Therefore, to narrow the differential diagnosis, it is necessary to take a full patient anamnesis, perform a focused physical examination and order laboratory tests. In this pictorial essay we review, illustrate and discuss, from a pathophysiological perspective, congenital, traumatic, and vascular diseases of the cranial nerves.

## Abstract (pt)

Os nervos cranianos representam extensões das estruturas funcionais do cérebro que cruzam a cabeça e o pescoço. Eles estão ligados a diferentes estruturas cranianas e se relacionam com diversas doenças. Uma compreensão profunda de sua complexa anatomia e aparência normal de imagem permite ao examinador identificar e caracterizar com mais precisão as anormalidades. A ressonância magnética com contraste é uma importante ferramenta para avaliar essas estruturas, especialmente as sequências tridimensionais de precessão livre de estado estacionário, que proporcionam alta resolução espacial e de partes moles, apesar da fina espessura dos nervos cranianos. Na maioria dos casos, os achados de imagem são inespecíficos, e uma anamnese completa, exame físico dirigido e testes laboratoriais são necessários para estreitar o diagnóstico diferencial. Este ensaio iconográfico ilustra e discute doenças congênitas, traumáticas e vasculares dos nervos cranianos, mostrando imagens em uma abordagem fisiopatológica.

## INTRODUCTION

The cranial nerves originate from the encephalon as twelve pairs, representing extensions of the functional structures of the brain and traversing the head and neck. They are numbered from I to XII in the craniocaudal direction. Cranial nerve I (the olfactory nerve) and cranial nerve II (the optic nerve) arise respectively from the cerebral hemispheres and the diencephalon, whereas cranial nerves III to XII all emerge from the brainstem. The cranial nerves pass through cranial bone foramina, exhibiting complex anatomy on their way, are enveloped by the leptomeninges, and are myelinated by Schwann cells, except for cranial nerves I and II, which are encased in olfactory ensheathing cells and oligodendrocytes, respectively.

Although radiological findings are nonspecific in many cranial nerve pathologies, magnetic resonance imaging (MRI) with heavily T2-weighted three-dimensional (3D) steady-state free precession (SSFP) sequences, which have high spatial resolution and provide good contrast between cerebrospinal fluid and the cisternal segments of the cranial nerves, and contrast-enhanced T1-weighted sequences, facilitates the depiction of abnormalities. Computed tomography (CT) scans with bone window settings are also quite useful for detecting cranial nerve pathologies, such as: agenesis/hypoplasia of the facial nerve canal and the geniculate ganglion fossa; fractures that extend through the skull base foramina in the context of trauma; enlargement or erosion of the skull base foramina caused by slow- or fast-growing cranial nerve tumors, respectively; and cranial nerve compression secondary to conditions that result in bone expansion and subsequent foraminal narrowing, including fibrous dysplasia.

Complete patient anamnesis, a focused physical examination and laboratory tests are crucial for making the clinical-radiological correlation, allowing radiologists to narrow the differential diagnosis. In this pictorial review, we briefly assess the normal anatomy of the cranial nerves, as well as illustrate and discuss some of the congenital, traumatic, and vascular diseases that affect them.

## OVERVIEW OF CRANIAL NERVE ANATOMY

I - Olfactory nerve (sensory function): passes below each frontal lobe via the olfactory sulci and transmits impulses from the olfactory epithelium to the brain.
II - Optic nerve (sensory function): emerges from each globe as a thick fiber bundle that passes through the optic canal, and merges with the other optic nerve to form the optic chiasm.
III - Oculomotor nerve (motor and parasympathetic functions): arises from the interpeduncular fossa of the midbrain; passes along the lateral wall of the cavernous sinus and through the superior orbital fissure; innervates the levator palpebrae superioris and most extraocular muscles (including the superior, medial and inferior rectus muscles, as well as the inferior oblique muscles); and has parasympathetic functions, controlling the ciliary and sphincter pupillae muscles.
IV - Trochlear nerve (motor function): arises from the dorsal surface of the midbrain just below the inferior colliculus, surrounds the contralateral surface of the midbrain, passes along the lateral wall of the cavernous sinus and through the superior orbital fissure, and innervates the superior oblique muscles.
V - Trigeminal nerve (motor and sensory functions): arises from the anterolateral surface of the pons, passes below the tentorium cerebelli, arriving at Meckel’s cave, where it splits into the ophthalmic, maxillary, and mandibular nerves, which pass through the superior orbital fissure, foramen rotundum, and foramen ovale, respectively, to exit the skull; enables mastication, carries proprioceptive and nociceptive afferents to the face and mouth, and innervates the tensor tympani, tensor veli palatini, and mylohyoid muscles, as well as the anterior belly of the digastric muscle.
VI - Abducens nerve (motor function): arises from the pontomedullary junction, near the midline; crosses the petrous apex within Dorello’s canal; ascends to pass through the cavernous sinus near the internal carotid arteries; exits through the superior orbital fissure and innervates the lateral rectus muscle.
VII - Facial nerve (motor, sensory, and parasympathetic functions): arises from the pons at the cerebellopontine angle; enters the internal auditory canals from an anterosuperior direction; passes through the temporal bones; exits through the stylomastoid foramen; controls facial expressions and taste sensation from the anterior two-thirds of the tongue and oral cavity; and regulates the function of the salivary glands (except the parotid gland), as well as that of the lacrimal, nasal, and palatine glands.
VIII - Vestibulocochlear nerve (sensory function): arise from the pons at the cerebellopontine angle; enters the internal auditory canals together with the facial nerve; splits into the cochlear nerve, in an anterior inferior direction, and the superior and inferior vestibular nerves, in a posterior direction; and carries afferents related to hearing and balance.
IX - Glossopharyngeal nerve (sensory, motor, and parasympathetic functions): arises from the medulla oblongata in the postolivary sulcus via a series of vertical rootlets lateral to the olivary body, above and along with those of the tenth and eleventh cranial nerves; carries sensory afferents to the oropharynx, posterior third of the tongue, carotid sinus, carotid body, and tympanic membrane; supplies motor innervation to the stylopharyngeus muscle; and provides parasympathetic innervation to the parotid gland.
X - Vagus nerve (sensory, motor, and parasympathetic functions): the longest of the cranial nerves, arising from the medulla oblongata via rootlets in the postolivary sulcus; exiting the posterior cranial fossa through the jugular foramen; descending in the carotid sheath posterior to the internal jugular vein and internal/common carotid arteries; and dividing into its pharyngeal, thoracic, and abdominal branches.
XI - Spinal accessory nerve (motor function): arises from caudal region of the nucleus ambiguus via rootlets in the postolivary sulcus, below and along with those of the ninth and tenth cranial nerves, joining the latter, from which it is indistinguishable.
XII - Hypoglossal nerve (motor function): arises from the medulla via a series of vertical rootlets in the preolivary sulcus; enters the hypoglossal canal of the occipital bone; descends to the neck near the carotid sheath; passes lateral to the hyoglossus muscle to enter the tongue from below; and innervates all intrinsic and extrinsic muscles of the tongue, except the palatoglossus muscle, which is innervated by the tenth cranial nerve.

## CONGENITAL DISEASES

### Septo-optic dysplasia

**Figure 1:**
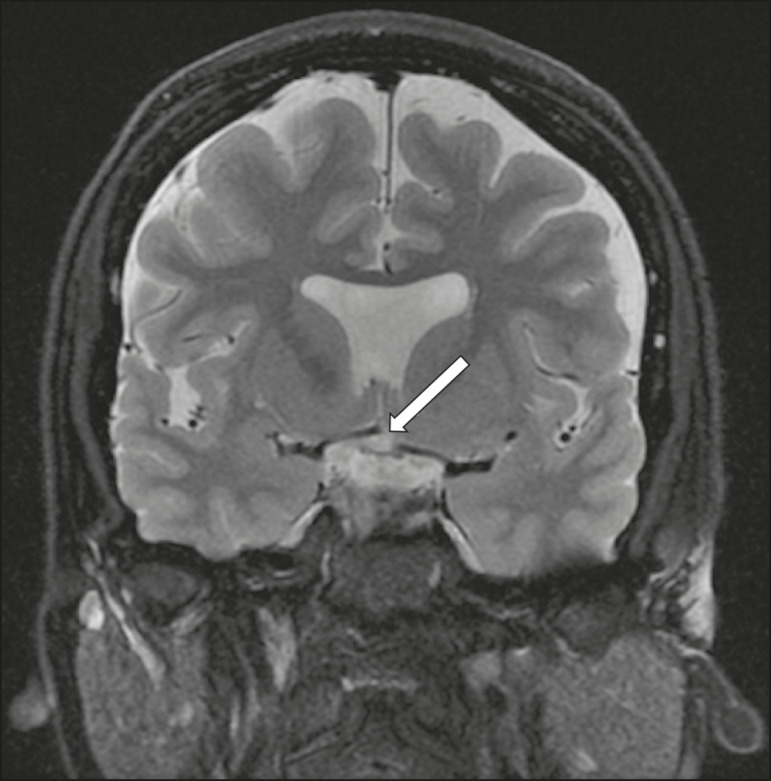
Septo-optic dysplasia. A 30-year-old female with headache and constant dizziness. Coronal T2-weighted MRI scan showing absence of the septum pellucidum and hypoplasia of the optic chiasm (arrow).

**Figure 2:**
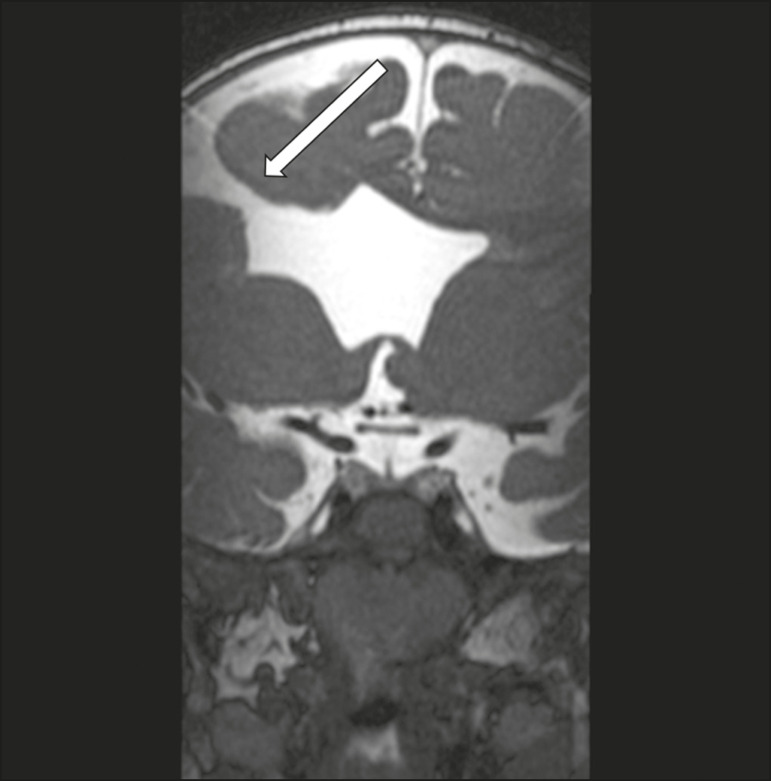
Septo-optic dysplasia plus. A 6-year-old male with eyelid myoclonus. Coronal T2-weighted MRI scan showing absence of the septum pellucidum and a wide cerebrospinal fluid-filled cleft (arrow) in the right frontal lobe (extending from the polar region to the medium convexity) and the anterior horn of the right lateral ventricle, wrapped in a thickened cortex. The right Sylvian fissure was flat and vertical, with thickened cortical segments, and there was, on the ipsilateral side, polymicrogyria of the insula and frontal operculum (not shown).

The septo-optic dysplasia spectrum comprises a heterogeneous group of rare disorders and malformations of the midline, including optic nerve hypoplasia and hypothalamic-pituitary dysfunction. Most patients have low visual acuity and nystagmus. Abnormalities include corpus callosum dysgenesis, absent septum pellucidum, cerebellar hypoplasia, and fornix aplasia (Figure 1). Most cases are segmental, although they can be complete. The involvement is bilateral in > 70% of cases. There can be pituitary hormone deficiency, ranging from deficiency of a single hormone to panhypopituitarism. The most common presentation is growth hormone deficiency^(1)^. As shown in Figure 2, the condition known as septo-optic dysplasia plus is characterized by septo-optic dysplasia accompanied by a malformation of cortical development, such as schizencephaly, heterotopy, and polymicrogyria^(2)^.

### Absence/hypoplasia of the abducens nerve

Absence or hypoplasia of the abducens nerve, as an isolated finding, is rare. There have been few reported cases of disturbances other than absence of abduction of the eye, which usually occurs in individuals with Duane or Moebius syndrome^(3)^.

### Duane syndrome

**Figure 3:**
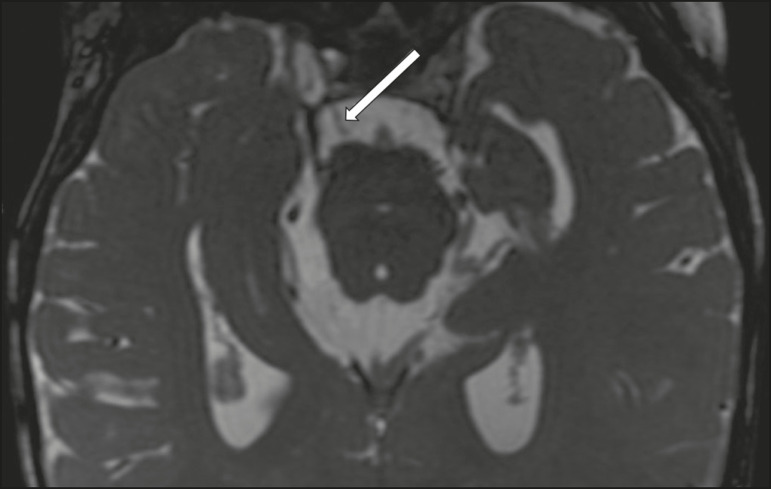
Absence and hypoplasia of the abducens nerve. A one-month-old male with congenital lateral rectus muscle palsy and globe retraction and presenting upshoot and downshoot with certain eye movements, therefore being diagnosed with Duane syndrome. Axial 3D-SSFP MRI sequence showing the right abducens nerve (arrow), whereas the left one is not visible.

Duane syndrome is a congenital condition, characterized by non-progressive, horizontal eye movement. It is caused by failure of the abducens nucleus and nerve to fully innervate the lateral rectus muscle^(3)^, as depicted in Figure 3.

### Moebius syndrome

**Figure 4:**
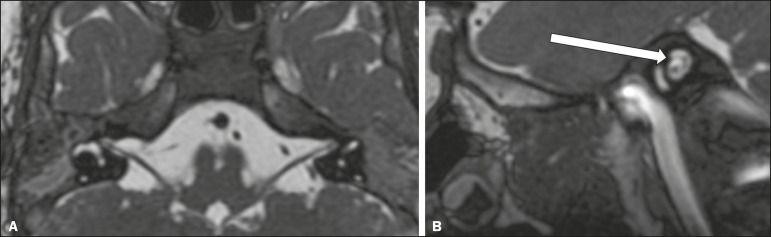
Moebius syndrome. Axial and sagittal 3D-SSFP MRI sequences (**A** and **B**, respectively) showing the absence of the facial nerve (arrow).

Moebius syndrome is a congenital condition characterized by paresis of the sixth and seventh cranial nerves. It has a multifactorial etiology, the causes including hypoxic insult, genetic susceptibility, and iatrogenic factors such as the use of prostaglandin-1 during pregnancy^(4)^. Individuals with Moebius syndrome can have craniofacial, cardiovascular and musculoskeletal defects, with or without other cranial nerve palsies, as well as abnormalities of the chest wall, spine, limbs, and soft tissues. Typical features of the syndrome include the following^(4)^: facial diplegia of the upper and lower facial muscles; impaired abduction of both eyes; craniofacial and limb malformations; hypoglossia; and long tract signs. Findings on CT and MRI (Figure 4) include various features^(4)^: pontine and medullary hypoplasia; absence of the medial colliculus; calcification in the region of the abducens nuclei; depression of the fourth ventricle; and absence of the hypoglossal prominence, suggestive of hypoglossal nuclei hypoplasia and cerebellar hypoplasia.

### Other conditions

There are many other congenital conditions involving the cranial nerves^(5)^, including Kallmann syndrome (agenesis or hypoplasia of the olfactory bulbs and sulci, together with hypogonadotropic hypogonadism); optic nerve hypoplasia; other eye movement disorders (congenital palsies of the third and fourth cranial nerves, double elevator palsy, and horizontal gaze palsy with progressive scoliosis); trigeminal neuropathies with oculoauriculovertebral dysplasia and hemifacial microsomia (Goldenhar syndrome); syndromes associated with inner ear malformations and vestibulocochlear nerve agenesis or hypoplasia (skeletal, craniofacial, and cervical syndromes); and less common combinations of syndromes (such as that of optic nerve hypoplasia and fifth cranial nerve dysfunction).

## TRAUMATIC DISEASES

Traumatic lesions of the cranial nerves are variable, depending on the length, course, and vascular supply of the nerve in question, as well its relationships with the surrounding bony and dural structures^(6)^. Possible mechanisms of cranial nerve trauma include stretching, impingement, and transection. Such trauma may be iatrogenic and sometimes requires surgical repair. A combination of CT and MRI is usually required for a thorough assessment, and neuropathies resulting from recent trauma can show enhancement on contrast-enhanced T1-weighted images. Of all the cranial nerves, the third is the most commonly injured, such injury typically being due to contusion (at the petroclinoid ligament), tearing, or complete avulsion (at the interpeduncular fossa).

### Olfactory bulb trauma

**Figure 5:**
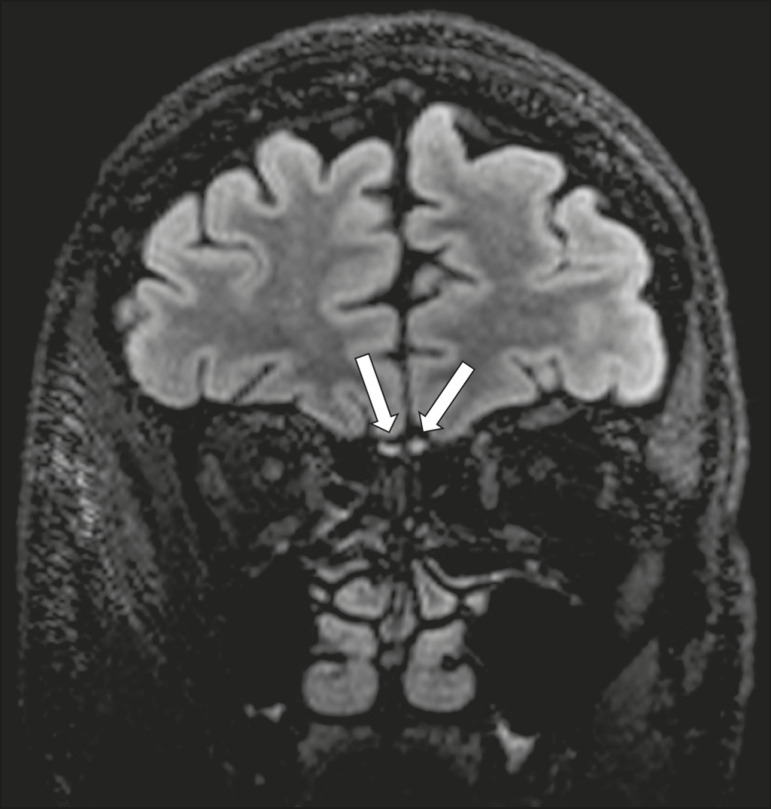
Olfactory bulb trauma. A 35-year-old male with anosmia after cranial trauma. Coronal fluid-attenuated inversion recovery MRI sequence showing a hyperintense signal in the olfactory bulbs (arrows).

Traumatic olfactory dysfunction occurs in 5% of cases of head trauma. During blunt trauma, movement of the brain relative to the cribriform plate causes shearing of olfactory nerve fibers^(7)^, as illustrated in Figure 5.

### Abducens nerve trauma

**Figure 6:**
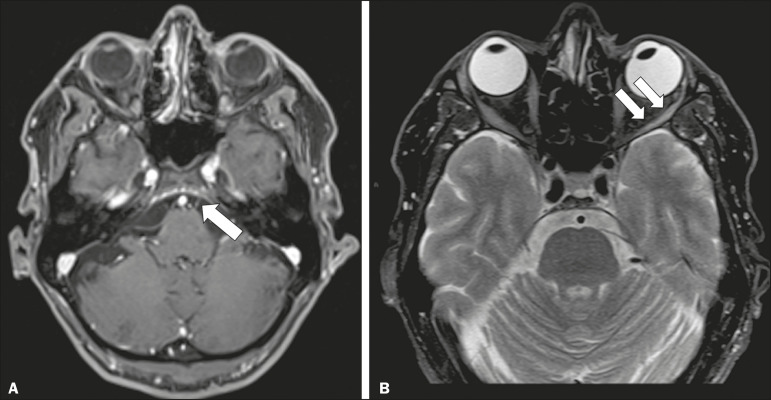
Abducens nerve trauma. A 50-year-old male with convergent strabismus after a motor vehicle accident. **A**: Axial contrast-enhanced T1-weighted spoiled gradient-recalled MRI sequence showing enhancement of the abducens nerve. **B**: Axial T2-weighted MRI scan showing a hyperintense signal, consistent with acute denervation, in the left lateral rectus muscle.

Isolated lesions of the abducens nerve due to trauma are rare and are usually associated with cranial or cervical fracture. The main sites are dural entry points at the petrous apex. As depicted in Figure 6, such lesions are caused by upward and downward movements of the brain, resulting from a linear force applied to the head^(8)^.

### Facial nerve trauma

**Figure 7:**
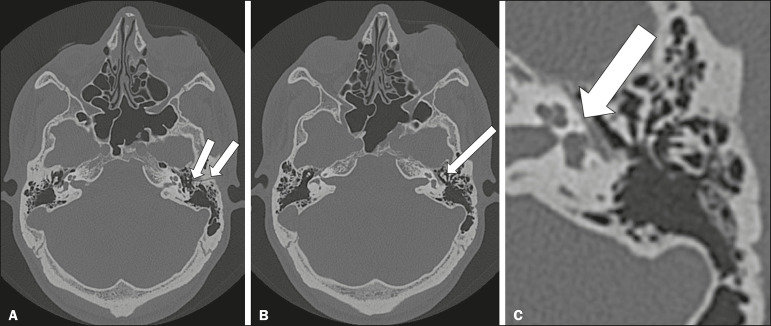
Facial nerve trauma. CT scans showing a transverse fracture of the left mastoid (arrows in **A**), resulting in incudomalleolar dislocation (arrow in **B**) and fracture of the canal of the tympanic segment of the facial nerve (arrow in **C**).

Facial nerve palsy is a common complication of temporal bone fractures (Figure 7). The sites most commonly affected are the labyrinthine segment, the geniculate ganglion and the tympanic segment. The complication may be immediate, due to partial or complete nerve transection or to compression of the nerve by a bone fragment, or delayed, due to nerve sheath edema/hematoma or compression by an expanding hematoma^(9)^.

## VASCULAR DISEASES

Vascular diseases can result in injury to the cranial nerves. Such injury can be caused by neurovascular compression, ischemic events, and hemorrhagic mechanisms, as well as by vascular malformations or neoplasms^(10)^.

### Aneurysms

**Figure 8:**
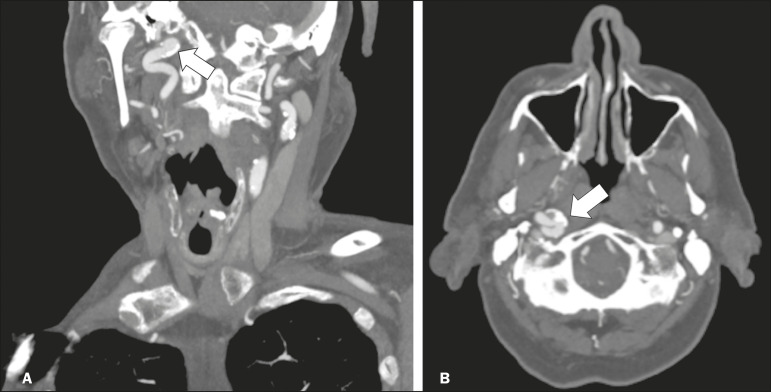
Cervical internal carotid artery aneurysm. An 82-year-old male with swallowing disturbance. CT angiography (coronal/sagittal in **A**; axial in **B**) showing a vascular loop with a partially thrombosed aneurysm in the right internal carotid artery (arrows in **A** and **B**). The lesion is along the course of the hypoglossal nerve.

**Figure 9:**
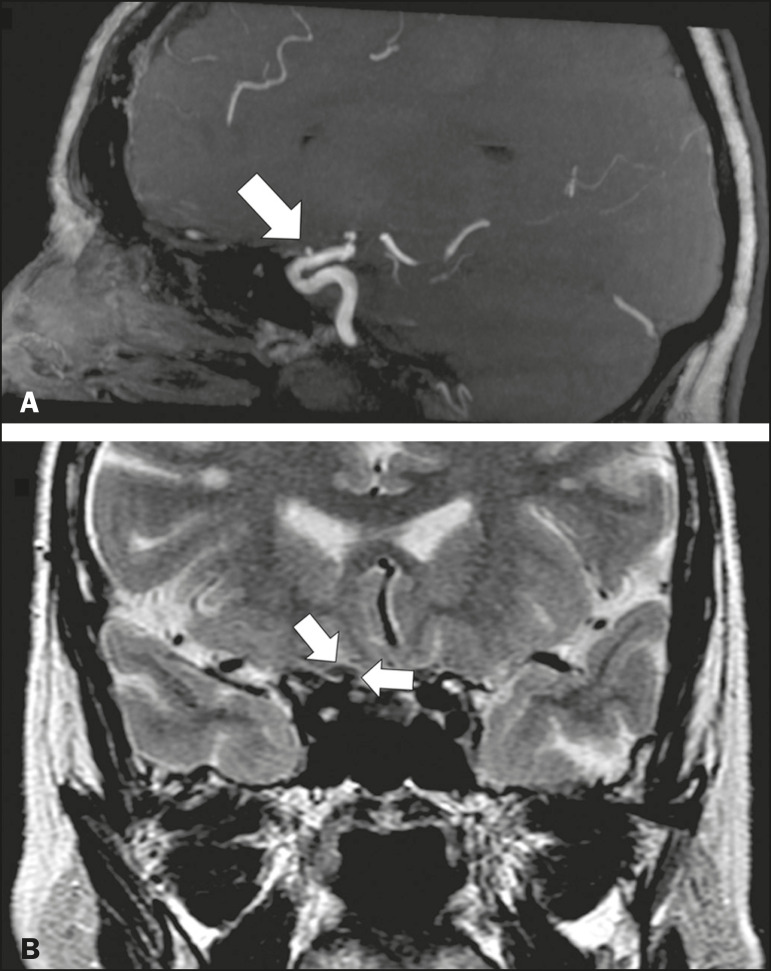
Ophthalmic artery aneurysm. A 65-year-old female with right temporal visual loss. Sagittal 3D time-of-flight magnetic resonance angiography (**A**) showing an aneurysm at the origin of the right ophthalmic artery (arrow). Coronal T2-weighted MRI scan (**B**) showing compression of the medial surface of the right optic nerve (oblique arrow) by the aneurysm (horizontal arrow).

Aneurysms can occur in different segments and may cause cranial nerve dysfunction due to compression and deformity. The nerves affected depend on the topography and orientation of the aneurysm^(10)^. The size of an aneurysm and its relationship to adjacent structures can be determined by CT angiography and magnetic resonance angiography (Figures 8 and 9, respectively).

### Trigeminal neuralgia

**Figure 10:**
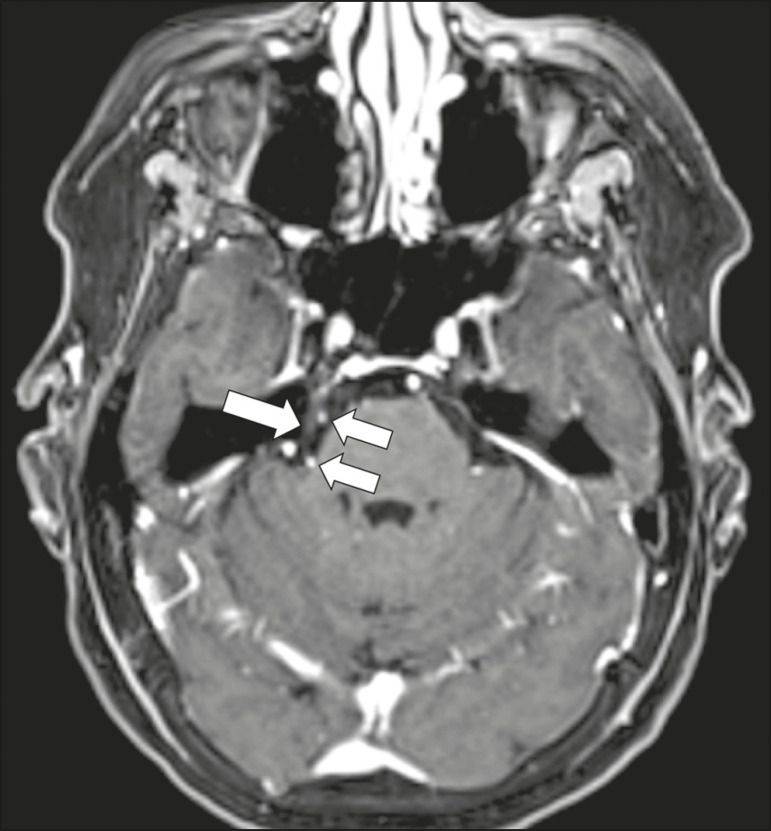
Neurovascular conflict. A 68-year-old female with right trigeminal neuralgia. Axial contrast-enhanced T1-weighted MRI scan showing a tortuous right superior cerebellar artery loop (medial arrows), tapering and displacing (laterally and inferiorly) the cisternal segment of the right trigeminal nerve (lateral arrow), including its origin at the pons.

Vascular compression of a cranial nerve, which can result in a neurovascular conflict, may be caused by a looped or bifurcating blood vessel. The thinner myelin in the transitional root entry zone, between the brainstem and the nerve, makes the site vulnerable to compression^(11)^. Most cases of trigeminal neuralgia are caused by abnormalities in the superior cerebellar artery (Figure 10), anterior inferior cerebellar artery, or basilar artery^(12)^.

### Carotid artery dissection

**Figure 11:**
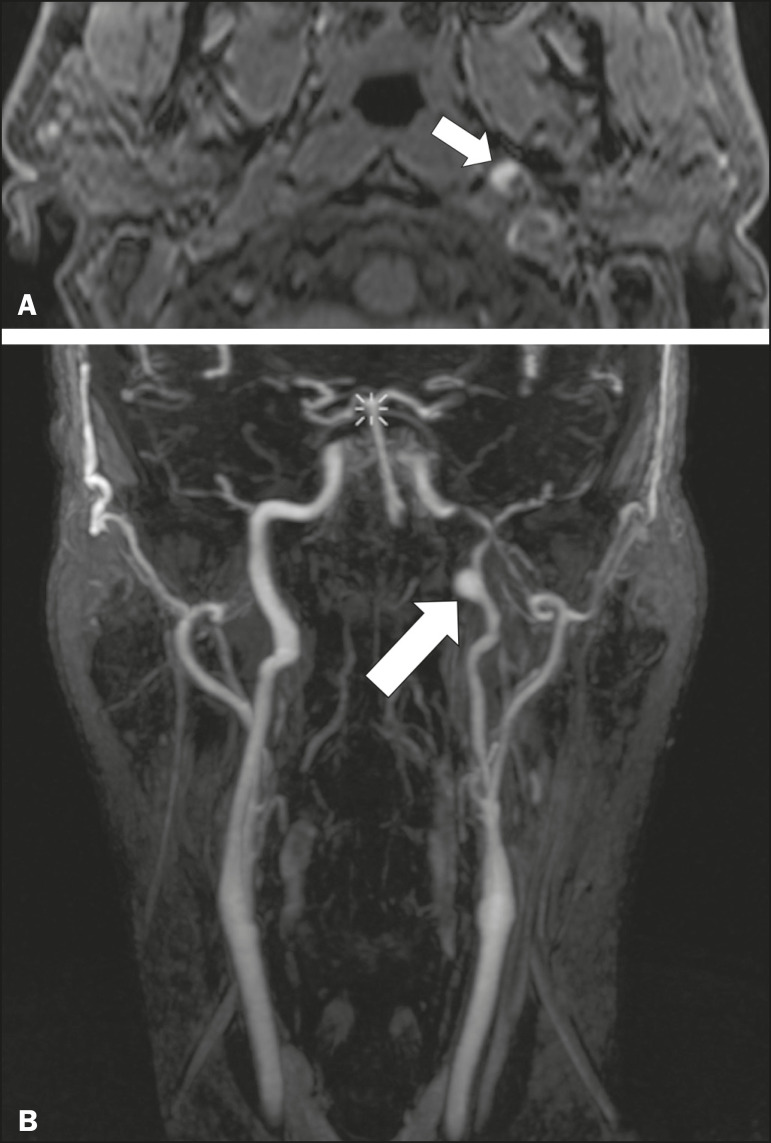
Carotid artery dissection. A 48-year-old male with sudden left cervical pain accompanied by gustatory changes. Axial T1-weighted fat-saturated MRI sequence (**A**) and coronal 3D time-of-flight magnetic resonance angiography (**B**) showing signs of recent dissection of the left internal carotid artery, depicting a crescent-shaped mural hematoma of methemoglobin, causing moderate to severe stenosis of the distal cervical segment. The accompanying focal dilation (0.7 cm) in the upper cervical segment is consistent with a pseudoaneurysm.

A tear in the intima of the carotid artery (carotid artery dissection) allows blood to flow into the subintimal and medial layers of the artery wall. In most cases, carotid artery dissection occurs spontaneously, although it may also occur after trauma or secondary to arteriopathy. In 30% of cases, there is pseudoaneurysmal dilatation (Figure 11), usually in distal cervical segments^(13)^. In subadventitial dissection, the medial muscle layer prevents narrowing of the lumen and the carotid artery wall expands. The lower (ninth, tenth, and twelfth) cranial nerves are close to the extracranial segment of the internal carotid artery in the upper cervical parapharyngeal space. Therefore, compression by the expanded artery may cause cranial nerve palsies. Involvement of the upper cranial nerves may occur when the dissection extends through the petrous and cavernous segments. Interruption of blood supply is another cause of cranial nerve palsy. The ninth and twelfth cranial nerves are supplied by the ascending pharyngeal artery, whereas the fifth and the seventh cranial nerves are supplied by the middle meningeal artery, branches of the external carotid artery^(14)^.

### Carotid-cavernous fistula

**Figure 12:**
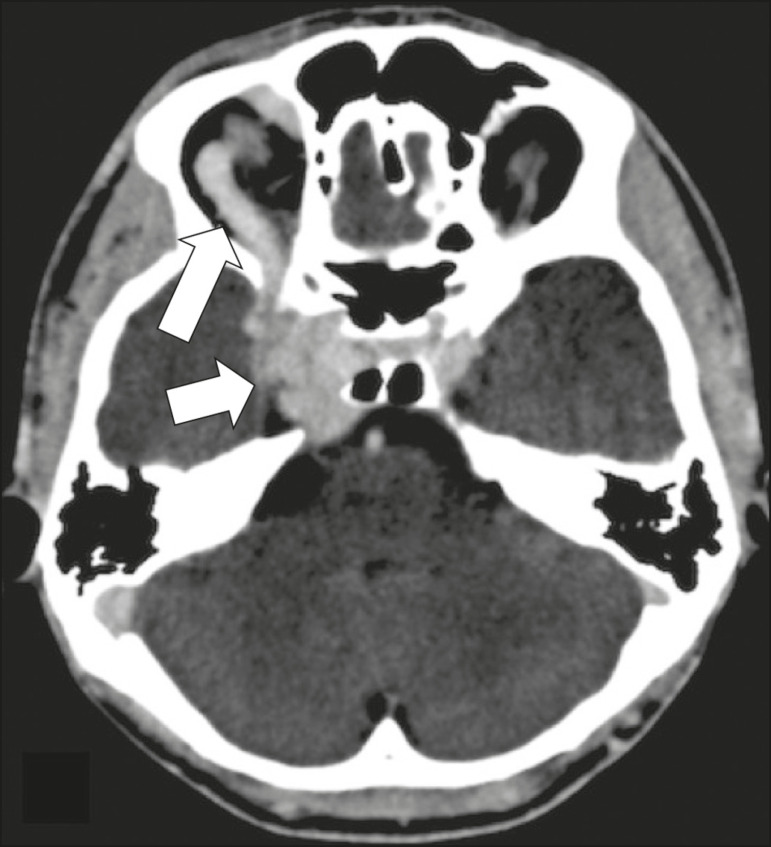
Carotid-cavernous fistula. Axial CT angiography showing dilation and early enhancement of the cavernous sinus and superior ophthalmic vein, resulting in right exophthalmia and convergent strabismus due to abducens nerve compression

Abnormal communication between the carotid artery and the cavernous sinus (carotid-cavernous fistula) may be classified anatomically as direct (type A); as a dural shunt (indirect) between the meningeal branches of the intracavernous internal carotid artery and the cavernous sinus (type B); as a dural shunt between the meningeal branches of the external carotid artery and the cavernous sinus (type C); or as a dural shunt between the meningeal branches of the intracavernous branches of the internal carotid artery, meningeal branches of the external carotid artery and the cavernous sinus (type D)^(15)^. Other classifications consider the etiology (traumatic or spontaneous) and hemodynamics (high or low flow) of the fistula. As can be seen in Figure 12, imaging findings include dilation and tortuosity of the superior ophthalmic vein; exophthalmos with enlargement of the extraocular muscles; an enlarged cavernous sinus; and intraorbital or periorbital edema. The most common symptoms related to the cranial nerves are diplopia and ophthalmoplegia (palsy of the third, fourth, or sixth nerve).

### Venous thrombosis

**Figure 13:**
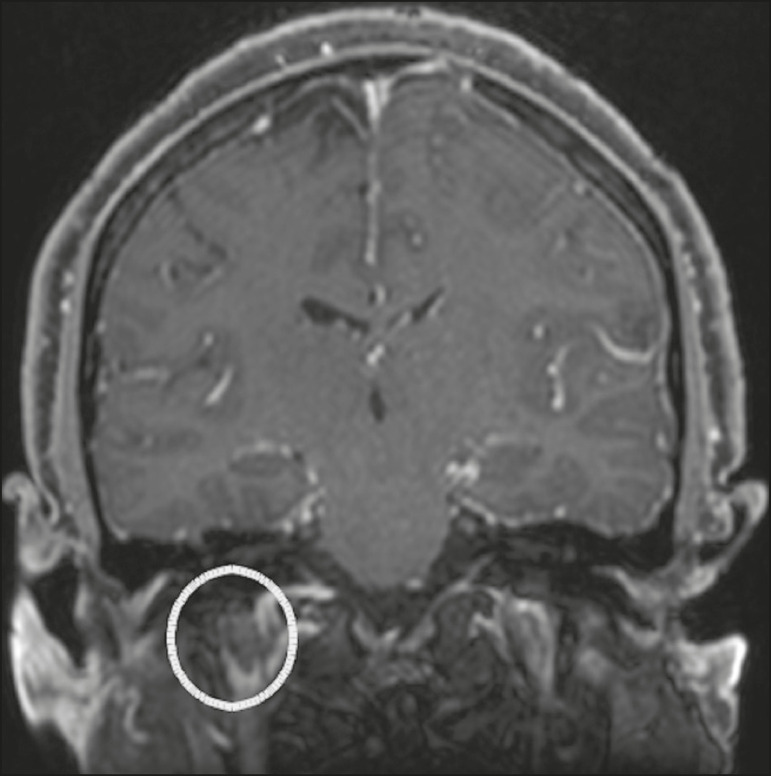
Venous thrombosis. A 36-year-old male presenting with dysphagia, dysphonia, and right-sided neck pain. Coronal contrast-enhanced T1-weighted MRI scan showing a focal thrombus within the right jugular bulb and the cranial segment of the internal jugular vein, near the jugular foramen, along the course of the ninth through twelfth cranial nerves (circle), which explained the symptoms.

Cerebral venous thrombosis may present a wide spectrum of signs and symptoms. Although uncommon, such thrombosis is associated with various types of cranial nerve palsy and should therefore be considered in their differential diagnosis, especially in patients with hypercoagulable conditions. Several cranial nerve syndromes have been attributed to extension of the clot into contiguous venous tributaries, with subsequent paralysis of the adjacent nerves. The involvement of the jugular foramen may cause syndromes of lower cranial nerves, characterized by several eponyms, such as Collet-Sicard syndrome^(16)^, which manifests as unilateral dysfunction of the ninth through twelfth cranial nerves, and Villaret’s syndrome, which is distinguished from Collet-Sicard syndrome by the fact that it is accompanied by Horner’s syndrome (Figure 13).

## CONCLUSION

The cranial nerves are connected to several different structures of the head and neck, and they can be affected by a variety of pathologies. Despite the fact that MRI provides excellent soft-tissue resolution and represents a very important tool to evaluate these structures, the imaging findings are nonspecific in most cases. Knowledge of anatomy and correlating imaging findings with clinical data are essential to reaching an accurate diagnosis.
